# Review of the Pig-Adapted African Swine Fever Viruses in and Outside Africa

**DOI:** 10.3390/pathogens11101190

**Published:** 2022-10-16

**Authors:** Mary-Louise Penrith, Juanita Van Heerden, Livio Heath, Edward Okoth Abworo, Armanda D. S. Bastos

**Affiliations:** 1 Department of Veterinary Tropical Diseases, Faculty of Veterinary Science, University of Pretoria, Onderstepoort, Pretoria 0110, South Africa; 2 Transboundary Animal Diseases, Onderstepoort Veterinary Research, Agricultural Research Council, Pretoria 0110, South Africa; 3 Biosciences, Animal and Human Health Program, International Livestock Research Institute (ILRI), Nairobi 00100, Kenya; 4 Department of Zoology and Entomology, Faculty of Natural and Agricultural Sciences, University of Pretoria, Pretoria 0028, South Africa

**Keywords:** African swine fever, genotypes, pig-adapted

## Abstract

The region in eastern, central and southern Africa (ECSA) where African swine fever (ASF) originated in a sylvatic cycle is home to all the *p72* genotypes of ASF virus identified so far. While 20 of the 24 genotypes have been isolated from outbreaks in domestic pigs in the region, only five of the genotypes (I, II, VIII, IX, X) have an extended field presence associated with domestic pigs. Of the genotypes that appear to be strongly adapted to domestic pigs, two have spread beyond the African continent and have been the focus of efforts to develop vaccines against ASF. Most of the experimental ASF vaccines described do not protect against a wider spectrum of viruses and may be less useful in the event of incursions of different strains or where multiple genotypes co-exist. The other three pig-adapted strains that are currently restricted to the ECSA region might spread, and priority should be given to understanding not only the genetic and antigenic characteristics of these viruses but also their history. We review historic and current knowledge of the distribution of these five virus genotypes, and note that as was the case for genotype II, some pig-associated viruses have the propensity for geographical range expansion. These features are valuable for prioritizing vaccine-development efforts to ensure a swift response to virus escape. However, whilst ASF vaccines are critical for high-production systems, global food security relies on parallel efforts to improve biosecurity and pig production in Africa and on continued ASFV surveillance and characterisation in the ECSA region.

## 1. Introduction

**Figure 1:**
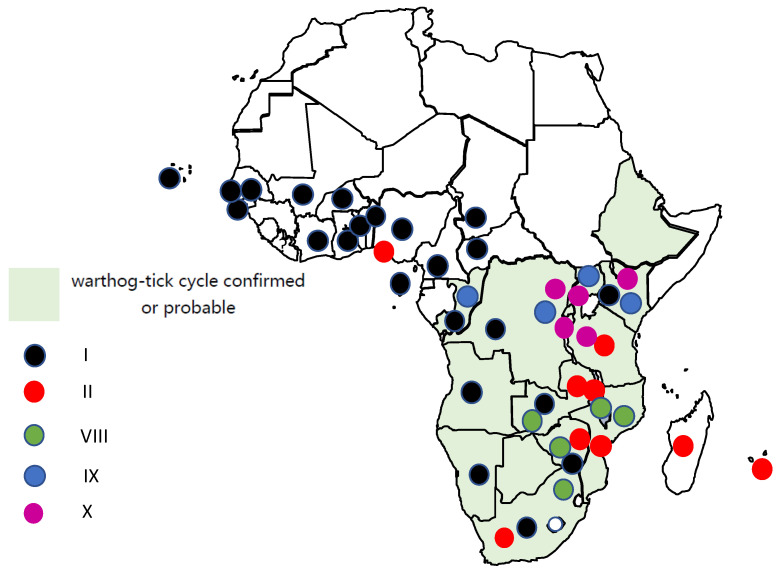
Map of the historic and current distribution of the five pig-adapted *p72* genotypes in Africa.

African swine fever (ASF) has been known in Africa for a century since its first published description by Montgomery in 1921 [1]. Two incidences of the virus escaping from Africa resulted in the spread of the disease in other continents and caused it to become a disease of global concern. The known hosts and countries in Africa that have been affected by the 24 *p72* genotypes are summarised in Table 1. The 5 pig-adapted viruses (I, II, VIII, IX, X) are listed first, followed by the remaining 19 genotypes, many of which are known from only a single country. The distribution of the five pig-adapted genotypes including their historic incursions in Africa is illustrated in Figure 1. 

The purpose of this review is to highlight, based on their history and current epidemiological distribution, the propensity of viruses belonging to five pig-adapted genotypes to spread across wider areas. We provide a summary of the available information about the origins and spread of genotypes I and II, and review the history of genotypes VIII, X and XI. Due to the global ASF situation, the widespread genotype II virus and to a lesser extent genotype I viruses are priority vaccine targets, but other pig-adapted viruses may be a cause of concern for the future spread in and outside Africa and have already caused the considerable loss of domestic pigs in Africa [56,57,58,59].

Two aspects that are not covered in the review are studies on virulence of ASFV isolates and publications relating to vaccine development, which are numerous, and most of which have involved genotype I or II viruses. Like other DNA viruses, ASF virus (ASFV) is stable genetically and biologically. However, studies based on the central variable region of the virus (CVR) and other regions containing tandem repeat arrays have enabled the distinction of subgroups that are more informative in terms of, for example, relatedness of ASF outbreaks [13,14,21,60,61,62,63,64].

## 2. Genotype I

Until 2007, genotype I was the most widespread ASFV and the only strain to have spread and become established for varying periods of time beyond the borders of Africa [3,21]. It remains the most widespread genotype in Africa. (Table 1; Figure 1). The first outbreaks of ASF in Europe involved pig farms close to Lisbon airport in 1957 and were attributed to pigs accessing galley waste from the airport, containing infected pork that originated from Angola [65,66]. The outbreak was rapidly eradicated, but a second incursion occurred in 1960, with rapid spread to Spain and subsequent incursions into France, Belgium, The Netherlands, Italy, Malta, the islands of Cuba and Hispaniola, shared by Dominican Republic and Haiti, and Brazil [67,68,69]. By 1995, ASF had been eradicated from all the affected countries apart from the Italian island of Sardinia, which is at the time of writing in the final stages of eradication [70,71,72,73]. An isolated outbreak occurred in Portugal in 1999 after pigs were introduced into a shelter infested by *Ornithodoros erraticus* ticks, which are able to maintain viable virus for long periods [74].

At approximately the same time as the second incursion into Portugal in 1960, outbreaks of ASF occurred in Senegal, with anecdotal evidence of outbreaks having occurred in Guinea-Bissau and Cape Verde as well [75]. Molecular studies based on restriction fragment length polymorphism (RFLP) indicated that the second incursion into Portugal occurred independently of the first based on small differences in the isolates obtained [76]. This distinctiveness was subsequently confirmed through CVR characterisation [21]. Incursions of ASF into São Tomé e Principe in 1978 [2] and Cameroon in 1982 [77] were caused by viruses that were indistinguishable from those circulating in Europe and the Caribbean at the time. Whilst it was initially not possible to determine whether they had originated from western Africa or elsewhere, CVR sequencing subsequently demonstrated that viruses from Brazil (1979) and Cameroon (1982) were identical [78]. However, it rapidly became endemic in the southern half of Cameroon where the majority of the pigs in the country were being produced [9,79,80]. Absence of *Ornithodoros moubata* complex ticks from the main pig-producing areas was confirmed [81], indicating that persistence depended on circulation in domestic pigs. The first outbreaks in northern Cameroon were reported in 2010 [82]. A molecular genetic study of outbreaks from 2010–2018 revealed three variants of genotype I, one of which was identical to the 1982 introduced virus [83], indicating a field presence of 28 years.

The first record of genotype I in domestic pigs in South Africa was reported in 1985 when an outbreak of ASF occurred on an isolated farm in the Limpopo province [4]. The outbreak was controlled by culling all the pigs on the affected farm. No further outbreaks of ASF caused by genotype I were reported in South Africa until 2016, when several outbreaks of the disease occurred in the central parts of the country. Subsequent outbreaks of ASF in South Africa have involved genotype I viruses that belong to three discrete subgroups identified by CVR sequencing, which predominantly affected smallholder pig farmers in peri-urban areas [6,84].

In 1996 ASF appeared for the first time in Côte d’Ivoire, and although apparently eradicated within months, outbreaks occurred in Benin, Togo and Nigeria towards the end of 1997, in Ghana in 1999 and in Burkina Faso in 2003 [75]. These events prompted a study that proposed *p72* genotyping to enable rapid PCR-based sequencing of clinical and formol-inactivated samples [3]. The virus that caused the 1957 incursion into Portugal was found to be genetically identical to viruses sampled in 1970 and 1972 from Angola across the CVR [21]. To date, the few viruses from Angola that have been typed belong to genotype I and it remains the most frequently encountered genotype in the DRC [27]. An investigation indicated that ASF viruses isolated from outbreaks in western Republic of Congo belong to the same subgroup of genotype I as the viruses from Angola, DRC and Europe [10]. Reported outbreaks from Namibia have also mainly involved genotype I [7,85].

A study of a large number of viruses of various genotypes based on the CVR of the *9RL* open reading frame revealed inter- and intra-genotypic variations, expanding upon the earlier study of Irusta et al. [78], suggested that more sensitive determination of relationships of variants within genotypes was possible [63]. This study indicated that the great majority of viruses isolated from outbreaks in Côte d’Ivoire, Benin, Ghana and Nigeria differed from those isolated from outbreaks in Senegal, Angola and Europe, although a single variant isolate from Côte d’Ivoire in 1996 clustered with the latter group [63]. The 1996 outbreak viruses differed from those circulating at the same time in Cameroon [63]. Further refinement of the technique enabled the identification of multiple subgroups within genotype 1 [21]. A subsequent study of isolates from outbreaks in Burkina Faso, Mali and Senegal from 1989–2016 demonstrated that while all the viruses belonged to genotype I, the isolates from Burkina Faso and Mali clustered with genotype Ia, while the Senegal viruses clustered in genotype Ib [11]. The study confirmed the close relationship between Senegal viruses and the single variant from Côte d’Ivoire identified previously [11,63]. An incursion into Côte d’Ivoire in 2014 via the port of San Pedro was caused by a virus that was most closely related to viruses circulating at the time in Cameroon [8]. CVR characterisation further revealed the presence of six discrete CVR variants in Nigeria, clustering within a monophyletic lineage, with only one of the six variants being transboundary, and identical to the Benin 1997 outbreak strain [13].

Despite its propensity to infect domestic pigs, genotype I viruses have also been associated with the sylvatic cycle. Two genotype I viruses were isolated from bushpigs in Kenya in 1961 and seven from *Ornithodoros* ticks sampled from warthog burrows in Livingstone National Park in Zambia from 1982–1983 [5]. The historical bushpig viruses remain the only report of genotype I in Kenya to date [5] and the only report of ASF viruses isolated from naturally infected bushpigs. The recovery of five genotype I virus variants from ticks from a single sampled park in Zambia is contrary to the low levels of genotype I variation associated with pig viruses. Two distinct clusters of genotype I viruses were subsequently isolated from *Ornithodoros moubata* complex ticks collected from warthog burrows in Mosi-oa-Tunya National Park in Livingstone district of Zambia’s southern province [86]. Some of these viruses were identical to isolates from outbreaks that occurred in domestic pigs in 2013–2015 in the Southern province [86]. The full genome sequences of two historical genotype I tick viruses from Livingstone National Park in Zambia were published in 2020 [12,87]. Four viruses genetically similar to the genotype I viruses that caused ASF outbreaks in South Africa were isolated from ticks collected in Kruger National Park two years prior to the outbreak in 1985. A fifth virus isolated from ticks collected in the Mkuze National Park also clustered within genotype I [4].

After three decades of a relatively constrained distribution to Sardinia and Africa, two non-haemadsorbing genotype I viruses of low virulence emerged in Henan and Shandong provinces in China in 2021 [88]. These viruses were shown to share a close identity to two genotype I viruses of lower virulence, OURT88/3 and NH/P68, which emerged in Portugal in the last century and have been described as vaccine candidates [88,89,90].

## 3. Genotype II

### 3.1. Genotype II in Africa

The molecular delineation of genotype II was based on *p72* C-terminus sequencing of a virus that was isolated from the first outbreaks of ASF to be experienced in Madagascar in 1998, where ASF subsequently became endemic [3,91]. However, following the introduction in 1997 from Africa [92,93], a virus identical across the C-terminal *p72* gene region was isolated from an outbreak in quarantined pigs in Mozambique in late 1998 [17]. This virus was subsequently identified in pig outbreaks that occurred in Zambia and Mozambique in 1993 and 2002, respectively [5]. A retrospective study of ASF viruses isolated in Zambia indicated that a genotype II virus caused outbreaks near Lusaka in 1993 and that an isolate from Mbala in 2013 shared high similarity to the Georgia 2007 outbreak virus [14]. A protracted presence of genotype II in Zambia is supported by the finding of a variant in an outbreak in Chipata in the endemic Eastern Province, which differed markedly at subgenotypic level from the Mbala and Lusaka viruses [14,64].

After its discovery in the Angónia district of Tete province, Mozambique in 1998 [17], the genotype II virus spread widely in Mozambique, causing outbreaks in Nampula and Cabo Delgado provinces in 2001–2004 as well as around the city of Maputo from September 2004 [94]. It was several years later that it was demonstrated that the majority of viruses isolated from *Ornithodoros* ticks from warthog burrows in the Gorongosa National Park, Mozambique belonged to genotype II [33], suggesting that genotype II viruses are maintained in the warthog-tick sylvatic cycle as well as in domestic pigs.

In 2007, the island of Mauritius suffered an incursion of a genotype II virus that was thought likely to have been introduced from Madagascar [19], based on the identity of the virus and the considerable amount of traffic of people and goods between the two countries. The last outbreak of ASF in Mauritius occurred in July 2008, and a self-declaration of freedom from ASF was submitted to WOAH on 23 April 2012 (https://rr-africa.woah.org/en/news/mauritius-declares-itself-free-of-asf/, (accessed on 1 September 2022)).

ASF outbreaks in Tanzania were reported to be sporadic until the turn of the century, when a large outbreak occurred in 2001 in Mbeya region, with spread to Dar-es-Salaam region [95]. These outbreaks were caused by a genotype XV virus, but since 2010 genotype II viruses have predominated in the southern and central regions of Tanzania [96]. A highly virulent genotype II virus that was identical to the Georgia 2007 virus caused a major outbreak in the Mbeya region in 2010, associated with a concurrent outbreak in the adjacent Karonga district of Malawi for which no molecular data were available [20]. However, viruses isolated from outbreaks in the Karonga district of northern Malawi in 2019 proved to belong to genotype II [18]. The infection persisted in Mbeya region and spread to several other regions including Dar-es-Salaam through illegal movement of pigs and swill feeding [18]. The virus has maintained a presence in south-western Tanzania and was isolated from symptomatic outbreaks in pigs that occurred in Dar-es-Salaam region in 2015 and Morogoro and Pwani regions in 2017 [96,97], as well as from asymptomatic pigs in 2011 [98]. The first full genome sequence of an African genotype II virus, which was isolated from an outbreak in pigs in Rukwa (Tanzania/Rukwa/2017/1) was published in 2021 [99]. 

Until 2015, reports of ASF outbreaks in domestic pigs in Zimbabwe were rare and involved isolated outbreaks in 1961, 1990 and 1992 [100], which were probably associated with warthogs. After no reports from 1992 to 2014, an outbreak occurred in free-roaming local breed pigs in Mashonaland Central province in the Mount Darwin area that shares a border with Tete province in Mozambique. The spread of infection was ascribed to salvage slaughter of infected pigs and the sale of infected pork, movement of infected pigs between villages and improper disposal of carcasses [100]. In 2019, further ASF outbreaks occurred in similar free-ranging pig populations, the first in Manicaland province, which shares a border with Manica province in Mozambique, in January, and in August and September again in Mashonaland Central province [38].

Outbreaks of ASF associated with a genotype II virus in domestic pigs outside the ASF controlled area have occurred in four provinces in South Africa since 2019 [84,101,102]. The genotype II virus was first reported in the North-West province and subsequently spread to the adjacent Gauteng province as well as the Eastern Cape and Western Cape Province. The source of these outbreaks and the transmission pathways remain unclear, but phylogenetic analyses suggest that genotype II viruses circulating in South Africa are most closely related to viruses from Mozambique and Zimbabwe (unpublished data, Transboundary Animal Diseases, Onderstepoort Veterinary Research, Agricultural Research Council, South Africa).

### 3.2. Genotype II Goes Global

In June 2007, ASF was confirmed in the Republic of Georgia, and the virus was found to share close similarity with the genotype II viruses from Madagascar and Mozambique as well as a virus isolated from an ASF outbreak in Lusaka, Zambia in 1991 [39]. By the end of 2007 the virus had spread to neighbours Armenia and Russia and had been reported from the autonomous republic of Abkhazia [39,103] and Nagorno Karabagh, a region disputed between Armenia and Azerbaijan [39]. In January 2008, an outbreak occurred in the village of Nic in north-western Azerbaijan, which was exceptional in having a predominantly Christian population that kept pigs [40]. In December 2008 and January 2009 infection was detected in wild boars in Iran, where there are no domestic pigs [46]. The infection in Russia was initially detected in wild boar carcasses in the Republic of Chechnya on the border with Georgia, and in 2008 and 2009 spread through the Russian Caucasus region, mainly through wild boars infecting each other and free-ranging backyard pigs [103]. From 2010 ASF spread northwards through western Russia, with some spectacular long-distance leaps to distant northern destinations. These were ascribed to movement of infected pork by the security forces, who fed it to pigs in swill without heat treatment [103]. After becoming established in western Russia, in March 2017 the first outbreak in Siberia was reported, involving a genotype II virus that was more similar to the original Georgia/2007 strain than the viruses currently circulating in Russia [104]. The disease has subsequently spread further east, reaching the Far East region of Russia by 2019, with outbreaks reported in both wild boars and domestic pigs [105]. An evaluation of the potential for wild boars to be responsible for trans-Siberian movement of ASFV indicated that this is unlikely, as the distances to be covered within the period are much too large [106].

In July 2012, the first genotype II outbreaks of ASF in Ukraine were reported [107], and outbreaks in wild boars and mainly backyard pigs continue to be reported to the World Organisation for Animal Health (WOAH, formerly OIE). Belarus reported outbreaks in domestic pigs in 2013, and in 2014 ASF reached the European Union, with the finding of dead wild boars that tested positive for genotype II ASF in Poland, Lithuania, Latvia and Estonia [108,109,110,111]. For the first time it became evident that ASF can be maintained in wild boar populations without involvement of domestic pigs, as outbreaks of ASF in pigs in the Baltic states were not numerous in comparison to those in wild boars, in which circulation continues [112,113]. In Poland the infection has been maintained largely in the wild boar population but with a large number of outbreaks in domestic pigs. Human activities were identified as important for the spread and stronger biosecurity on pig farms was recommended [114]. Between 2014 and 2019 ASF remained restricted to the eastern districts of the country, but in 2019 the western districts also became infected, with both wild boars and pig farms involved [115].

The rapid escalation of the spread of genotype II ASFV in Europe from 2016 onwards was reflected in the flood of immediate notifications to WOAH as well as growing numbers of publications, starting with outbreaks in Moldova in 2016, followed by Romania and Czech Republic in 2017, Bulgaria, Hungary and Belgium in 2018, Serbia, Slovakia and Greece in 2019, Germany in 2020 and mainland Italy in 2022 [41,42,43,44,45]. The outbreaks in Moldova, Romania and Bulgaria were mainly in backyard pig farms, although there was involvement of some commercial pig farms and wild boar [116,117,118,119]. The first and only reported outbreak of ASF in Greece and the first outbreak of ASF in Serbia occurred in backyard farms [41,44]. North Macedonia reported its first outbreak in January 2022 (https://www.woah.org/app/uploads/2022/02/asf-report6.pdf, (accessed on 1 September 2022)). The outbreaks have been restricted to wild boar in Belgium, Czech Republic and Hungary [43,120,121] and both Belgium and Czech Republic are now officially free of ASF [122,123]. Dead wild boar infected with a genotype II ASFV were detected in northern Italy on 29 December 2021, the first occurrence of ASF on the Italian mainland since 1980 [38,42].

In August 2018, the first report of ASF emanated from China, home to almost half of the world’s pig population and an event that had been widely feared for some time [124,125,126]. The virus proved to be a genotype II virus most closely related to those circulating in eastern Europe and Russia [127,128]. The virus spread rapidly across China, having affected most of the 31 provinces by April 2019 [129,130,131]. The rapid spread of the disease was attributed to lack of a surveillance system capable of providing early detection of ASF, inadequate reporting of ASF, and feeding with unsafe feeds and swill [131]. The spread in Asia was rapid, starting with infection in Mongolia and Vietnam in January 2019 [132,133]. These outbreaks were followed by Cambodia, Philippines, China (Hong Kong), North Korea, Lao, Myanmar, South Korea, Timor-Leste and Indonesia [48,50,134,135,136] and reports to WOAH. In March 2020 Papua New Guinea became the first country in Oceania to report an outbreak of ASF to WOAH. India reported its first outbreaks in May 2020, in the north-eastern parts of the country where there are many smallholder pigs [137,138]. Malaysia reported its first outbreaks of ASF in February 2021, and in May 2021, Bhutan declared outbreaks in the Chhukha district, in scavenging pigs in a town close to the border of West Bengal in India [52,53]. Thailand officially reported ASF for the first time after Taiwan reported finding viable ASFV in sausages in a postal consignment from Thailand [139,140,141]. Taiwan has managed to remain ASF-free although in April 2021 the carcass of a pig infected with ASFV washed up on the coast of Walni district, New Taipei [142]. In May 2022, ASFV was confirmed in samples of pigs from Nepal, where unusual mortality among pigs had been occurring since March [54]. Where published information exists, genotype II viruses have been responsible for all the outbreaks: Mongolia [47], Indonesia [48], South Korea [143,144], Vietnam [49,51,145] and India [146,147].

With the exception of South Korea, where most of the outbreaks of ASF have occurred in wild boar, mainly in the demilitarised zone that borders on North Korea [148,149,150,151], the great majority of Asian outbreaks have occurred in domestic pigs. However, infection has been reported in wild boar in several other countries, including China [152], Vietnam and Lao [153] and most recently Hong Kong. Furthermore, ASF has caused severe mortality in the vulnerable and protected Asian bearded pigs in Malaysia, and the presence of the virus in India and Indonesia has raised concerns for all 11 wild pig species in Southeast Asia, all of which have small populations and limited distribution [154].

The genotype II virus finally reached the American region in 2021, when ASF was reported from the island of Hispaniola shared by Dominican Republic and Haiti [55]. This historical overview of genotype II demonstrates that homogeneity across the C-terminal *p72* gene region of pig outbreak strains in Africa over a number of decades is a typical feature of pig-adapted viruses. When considered retrospectively, it is now clear that this, together with the geographical range expansion on the African continent, inclusive of Indian Ocean islands that occurred in the decade prior to the 2007 introduction to Georgia, was arguably a strong signal that this was a genotype of potential global concern. These epidemiological features are therefore not only potential indicators of the next likely excursion, but also serve as a valuable guide on how best to prioritise research efforts, inclusive of vaccine development.

### 3.3. Genotype VIII

One of the first published genotype VIII viruses was based on an isolate from the same outbreaks in a quarantine facility in Mozambique as a genotype II virus, although co-infection with both viruses had not occurred [17]. This study confirmed a prolonged field presence of genotype VIII with links to domestic pig outbreaks in Zimbabwe, Zambia and Malawi from 1961–1998. A subsequent, expanded study revealed that genotype VIII viruses have long been associated with circulation in domestic pigs in Zambia and in the endemic area of Malawi [5,60]. Isolates from outbreaks in domestic pigs between 1982 and 1989 in the endemic area of Malawi as well as ticks inhabiting pig shelters in that area [155] proved to belong to genotype VIII [60], as did most of the viruses isolated from pig outbreaks in the Eastern province of Zambia, adjacent to the endemic areas in Malawi and Mozambique [14]. As is the case with genotypes I and II, genotype VIII viruses associated with domestic pig outbreaks are highly conserved across the *p72* genotyping region. CVR characterisation has similarly been shown to be useful for discerning discrete variants. In the study conducted by Lubisi et al. [60], only two genotype VIII variants were discernible based on C-terminal *p72* gene sequencing whereas the CVR phylogeny revealed the presence of seven discrete CVR variants. Of these, variant F was shown to have an extended transboundary field presence of 30 years (1961–1991), causing outbreaks in Zimbabwe, Zambia, Mozambique and Malawi [60]. Despite being one of the first genotypes to be extensively characterised [5,60,155], the full genome sequence of a genotype VIII virus isolated from a tick in Malawi only became available later [156,157]. The first ASF outbreak outside the endemic area of the Eastern Province in Zambia occurred in an isolated piggery in 1989 and proved to have been caused by a genotype VIII virus [14].

A genotype VIII virus was isolated from an outbreak of ASF in domestic pigs in the ASF controlled area in South Africa in 1995 [4], where ASF outbreaks were most often associated with the sylvatic cycle [158].

### 3.4. Genotype IX

Genotype IX and X viruses have an East African distribution, and are the predominant genotypes circulating in Kenya and Uganda [159]. Genotype IX ASFV also circulates in Tanzania [96,97,159] and has been reported from outbreaks in pigs in DRC [23,24] and the Republic of Congo [10].

The history of genotype IX started in Uganda. A Ugandan virus assigned to genotype IX [3] clustered with another Ugandan virus identified in 1989 as differing from the viruses circulating in Europe and western Africa by RFLP [76]. A further Ugandan virus was assigned to genotype IX in a study that included viruses from a number of countries in East Africa [5]. Full genome sequencing of five Ugandan isolates from outbreaks in two villages in 2015 indicated that four of the isolates were identical to each other and all were closely related to the only known sequence of a genotype IX virus [160].

Genotype IX virus isolates from outbreaks that occurred close to the border with Uganda in western Kenya in May 2006 proved to be identical to isolates from outbreaks in eastern Uganda in 2003 [161]. Outbreaks in pigs in western and central Kenya in late 2006 and 2007 with high mortality were identical to isolates associated with outbreaks that occurred in three districts in Central Uganda in 2007 [22]. Viruses isolated from ASF outbreaks in eastern Congo Republic in 2009 clustered with the latter Kenya and Uganda outbreak viruses [10]. A study carried out in western Kenya in 2011–2013 also yielded genotype IX viruses [162] that shared high similarity with eastern Ugandan viruses during the same period [61].

Although genotype IX is generally considered to be highly virulent [61,163], circulation in asymptomatic pigs has been reported from Uganda, Kenya and Tanzania [25,97,164,165]. Some of the reports refer to retrieval from apparently healthy slaughtered pigs and could simply represent pigs slaughtered in the incubation period, as panic sale of pigs when an outbreak is suspected by farmers is widely reported [164,165], but pig populations with increased resistance to ASF have been identified in western Kenya [162,165].

### 3.5. Genotype X

Like genotype IX, the uniqueness of a Tanzanian virus had been detected by RFLP [76], and it and related viruses from Tanzania, Burundi, Kenya and Uganda were assigned to genotype X [3]. The Tanzanian isolate assigned to genotype X came from a warthog [3]. Reports of ASF outbreaks in pigs in Tanzania were scarce until the turn of the century, when the 2001 outbreaks in Mbeya and Dar-es-salaam were followed by outbreaks in Arusha region in 2003 and Kigoma region in 2004 [95]. The *p72* genotyping was not used to convey the results of molecular typing, but the Kigoma outbreak viruses clustered with genotype X viruses and were suspected to have been introduced by refugees from Burundi [95]. Genotype X viruses were associated with outbreaks of ASF in the Longido district of the Arusha region in 2009 and in the Arusha and Kilimanjaro regions of Tanzania in 2013 [30,166].

Outbreaks in six districts of South Kivu province in DRC in December 2018 were associated with genotype X viruses that were similar to viruses reported from Kenya, Tanzania and Uganda [28]. Molecular characterisation of viruses from two of the districts in 2020 yielded four genotype X viruses and a single genotype IX virus, with no co-infection with the two genotypes [23]. The genotype X outbreak viruses were similar to those isolated from healthy pigs in South Kivu in a survey in 2016 [28]. The full genome sequence of the genotype X virus has been published [167].

In 2018, genotype X caused an ASF outbreak in the Rutana region, Burundi [29] There is little published information about ASF in Burundi, but both of the previous outbreak viruses (1984, 1990) belonged to genotype X [29].

Variability in the virulence of genotype X viruses has been noted, and it has been reported to be of lower virulence than genotype IX [163,168]. An experimental study using a tick isolate of a genotype X virus from Kenya considered to be moderately virulent indicated that manifestations in domestic pigs were dose dependent [169], which is probably true of ASF viruses in general.

## 4. Discussion

The affinity for domestic pigs of ASF genotypes other than those regarded as pig-adapted is unknown. Although 20 of the 24 known ASFV genotypes have been isolated from domestic pigs (Table 1), several of the infection events probably took place in areas with few pigs. In both Kenya and South Africa, most of the earlier reported outbreaks of ASF in pigs took place on isolated farms, without further spread [1,4,158], and would likely have been self-limiting if the pigs had not been culled. However, in long-time endemic areas in Angola, DRC, Kenya, Malawi and Mozambique a higher rate of survival of pigs has been reported, associated with a proportion of the pigs having developed innate resistance to the pathogenic effects of the virus [162,165,170,171,172,173,174]. Virus or viral DNA may be detected in healthy pigs for a variable length of time after infection, which is often asymptomatic, and infective virus may be present for up to 30 days in blood after infection with a highly virulent virus [175,176]. Infective virus may be present in tissues, in particular lymph nodes and tonsils, for longer [176]. The role of persistently infected pigs in maintenance of ASF infection is much debated, but there is increasing evidence that pigs that fully recover from ASF are inefficient at transmitting the virus through contact [94,177,178,179].

It is probable that availability of pig populations to enable further spread after an introduction of an ASFV facilitates adaptation to pigs, although this may take a long time. The warthog-tick cycle has not been investigated in Ethiopia, where a novel genotype of ASFV with two variants was reported for the first time in domestic pigs in 2011 and 2014 [37]. Despite the small pig population, the fact that the virus was able to circulate and spread over a period of at least three years may indicate that with the right opportunity any of the genotypes could become adapted to pigs.

Two further questions cannot be answered based on the information we have at present. One is whether genotypes VIII, XI and X could develop the same propensity for dramatic transcontinental spread demonstrated by genotypes I and II. The genotype II virus originally identified from an outbreak in pigs in a remote part of Mozambique has now gone global, yet the genotype VIII virus isolated from the same outbreak has a much more modest distribution, in both Mozambique and elsewhere [60,94]. It was isolated from a single outbreak in pigs inside the endemic area of South Africa [4] but has not spread further, while a genotype II virus has been responsible for a great many of the outbreaks outside the endemic area in South Africa since 2018 [101]. Genotypes IX and X predominate throughout East Africa but so far have not spread further, apart from limited spread in Central Africa [10,27].

The other question is whether pig-adapted *Ornithodoros* ticks might be involved in the adaptation of the viruses to pigs. This arises because genotype VIII viruses have been isolated from pig-adapted ticks in Mchinji District, Malawi [60,155]. Although a domestic pig-tick cycle has not been confirmed in the district of Mozambique where the genotype VIII virus was found in 1998, this district is adjacent to the Mchinji district in Malawi and it is highly likely that the cycle does exist there as well. Furthermore, neither a warthog-tick cycle nor a domestic pig-tick cycle has been investigated in Angola, where to date only genotype I virus has been found, but the existence of a sylvatic cycle at least historically is probable.

One of the strongest imperatives is to develop safe and effective vaccines against ASFV [56,59]. Although it is still substantially true that no vaccines are available for ASF, considerable progress has been made towards a commercial live attenuated vaccine (LAV) that is reported to be both safe and efficacious in preventing ASF caused by genotype II ASFV in Vietnamese pigs [180]. Some progress has been made in developing vaccines that protect against more than one genotype [89,181]. Given the large number of genotypes circulating in a large part of sub-Saharan Africa [182], it would be impractical to hope for a single cross-protective vaccine. However, a vaccine offering a broader spectrum of protection than just genotype II would certainly be advantageous in the medium to longer term [59]. Efforts described to develop a vaccine against genotype IX are encouraging [183,184].

In spite of a need to prioritise vaccine development for ASF, millions of poor households, especially in sub-Saharan Africa but also in the Asia-Pacific region and the Caribbean, keep pigs as a coping mechanism [58,134,136,185,186,187,188,189] or because of their cultural importance [190]. The utility of a vaccine in these settings is questionable, especially in the lower income countries where help from government is of necessity limited by lack of resources. The spread of the pig-adapted viruses has been possible due to sub-optimal management of pigs, such as feeding international waste as swill, trade in infected pigs or pork, and allowing pigs to scavenge in areas where infected waste food is likely to be available. In the 2015 ASF outbreak in Zimbabwe, it was reported that penned pigs survived, while all the free-roaming pigs succumbed to ASF [100]. A detailed description of how to apply biosecurity to informal pig farming systems and their associated value chains is beyond the scope of this review and has been published elsewhere [58,134,185,191,192,193,194,195], but biosecurity will always be of paramount importance in prevention of ASF even if a broad-spectrum vaccine should become available. Firstly, biosecurity should be in place wherever pigs are kept, because countries free of ASF are not likely to permit or maintain vaccination against it. Secondly, obtaining adequate coverage in short cycle species such as pigs kept in a dynamic informal sector is challenging because accurate information on numbers and distribution of pigs is usually lacking. Thirdly, many simple biosecurity measures are highly cost-effective compared to vaccination, taking into account both the cost of the vaccines and their distribution.

The genotype VIII, IX and X viruses all have a long history in Africa and are currently restricted to a few countries in that continent. However, they have shown a far greater tendency for geographical range expansion than most of the other ASFV genotypes. It is important to remember that genotypes I and II also originated in a sylvatic cycle in Africa and had a relatively long association with domestic pig outbreaks before escaping to other continents, and therefore surveillance and typing of outbreaks in Africa remains important for risk management of ASF.

## Figures and Tables

**Table 1:** Known hosts and countries in Africa affected by the 24 known *p72* genotypes: in the table ‘tick’ refers to *Ornithodoros moubata* complex ticks.

Genotype	Sylvatic Hosts	Domestic	Distribution (Past, Present)	References
**I ^a^**	Bushpig, warthog-associated tick, warthog	+++++	Africa: Angola, Benin, Burkina Faso, Cameroon, Congo Republic, Côte d’Ivoire, Chad, Central African Republic (CAR), Democratic Republic of Congo (DRC), Gambia, Ghana, Kenya, Mali, Namibia, Nigeria, São Tomé e Principe, Senegal, Sierra Leone (by extrapolation as diagnosis was based only on antibodies), South Africa, Togo, Zambia, Zimbabwe	[2,3,4,5,6,7,8,9,10,11,12,13,14,15]
**II ^b^**	Warthog-associated ticks in Mozambique	+++++	Africa: Madagascar, Malawi, Mauritius, Mozambique, Nigeria, South Africa, Tanzania, Zambia, Zimbabwe	[3,5,14,16,17,18,19,20]
**VIII**	Warthog-associated tick; pig-associated tick	+++	Malawi, Mozambique, South Africa, Zambia, Zimbabwe	[3,4,5,14,19,21]
**IX**	Warthog	+++	Congo Republic, DRC, Kenya, Uganda	[3,10,22,23,24,25,26,27]
**X**	Warthog; warthog- associated tick	+++	Burundi, DRC, Kenya, Tanzania, Uganda	[3,28,29,30]
III	Warthog-associated tick	+	Botswana, South Africa	[3,4,7,12,31]
IV	Warthog	+	South Africa	[3,4,32]
V	Warthog	++	Malawi, Mozambique	[3,19,33]
VI		+	Mozambique	[3,7,17]
VII	Warthog	+	Botswana, South Africa	[3,4,7]
XI	Warthog- associated tick	-	Zambia	[5]
XII	Warthog-associated tick	+	Malawi, Zambia	[5,7]
XIII	Warthog-associated tick	-	Zambia	[5]
XIV	Warthog-associated tick	+	DRC, Zambia	[5,14,27]
XV	Warthog-associated ticks	+	Tanzania	[5,34,35]
XVI		+	Tanzania	[5]
XVII		+	Zimbabwe	[7]
XVIII		+	Namibia	[7]
XIX	Warthog-associated tick	+	South Africa	[4,7]
XX	Warthog-associated tick; European wild boar (South Africa)	+	DRC, South Africa	[4,7,31,32,36]
XXI	Warthog, warthog-associated tick	+	South Africa	[4,7,36]
XXII	Warthog-associated tick	+	South Africa	[4,7,12,31,36]
XXIII	Not investigated	+	Ethiopia	[37]
XXIV	Warthog- and pig-associated ticks	-	Mozambique	[33]

^a^ Genotype I in Europe: Portugal, Spain, Italy, Sardinia, Malta, Netherlands, Belgium. Americas: Brazil, Dominican Republic, Haiti [38]. ^b^ Genotype II Transcaucasus: Armenia, Azerbaijan, Georgia. Europe: Belarus, Belgium, Bulgaria, Czech Republic, Estonia, Germany, Greece, Hungary, Italy, Latvia, Lithuania, Moldova, North Macedonia, Poland, Romania, Russia Serbia, Slovakia, Ukraine. Asia: Bhutan, Cambodia, China, India, Indonesia, Iran, Lao, Malaysia, Mongolia, Nepal, Philippines, North Korea, South Korea, Timor-Leste, Vietnam. Oceania: Papua New Guinea. Americas: Dominican Republic, Haiti [39,40,41,42,43,44,45,46,47,48,49,50,51,52,53,54,55].

## Data Availability

Not applicable.
